# Psychological aspects of hippotherapy for children with severe neurological impairment: An exploratory study

**DOI:** 10.1371/journal.pone.0320238

**Published:** 2025-04-08

**Authors:** Karin Hediger, Marina Lunzenfichter, Eva Markzoll, Wanda Arnskötter, Martina Schaudek, Gerhard Kluger

**Affiliations:** 1 Faculty of Behavioural Sciences and Psychology, University of Lucerne, Luzern, Switzerland; 2 REHAB Basel, Clinic for Neurorehabilitation and Paraplegiology, Basel, Switzerland; 3 Department of Epidemiology and Public Health, Swiss Tropical and Public Health Institute, Allschwil, Switzerland; 4 Research Institute for Rehabilitation, Transition, and Palliation, Paracelsus Medicine University, Salzburg, Austria; 5 Clinical Psychology and Animal-Assisted Interventions, Faculty of Psychology, University of Basel, Basel, Switzerland; 6 Specialist Center for Pediatric Neurology, Neurorehabilitation, and Epileptology, Schön Klinik, Vogtareuth, Germany; University of Pretoria, SOUTH AFRICA

## Abstract

**Background and objectives:**

Neuropediatric hospitalization presents significant psychological challenges that affect quality of life, learning, and treatment adherence. Hippotherapy might address these factors, but its psychological effects are underexplored. This paper explores the psychological and neuropsychological potential of hippotherapy in pediatric neurorehabilitation. Objectives include analyzing (1) patient characteristics, (2) session characteristics, (3) patients’ psychological and neuropsychological reactions, and (4) therapists’ perceptions of the extent to which the therapeutic goals were realized in the sessions.

**Methods:**

This retrospective explorative study examines data from 581 children and adolescents (M =  9.01 years, SD =  3.88) with severe neurological disorders who underwent hippotherapy. We used the registration form for each patient and session-documentation forms for each hippotherapy session. We extracted information regarding patient and session characteristics, performed a content analysis on the documented reactions of the patients and the predefined neuropsychosocial goals set by the rehabilitation team, and examined to what extent the goals were met during hippotherapy. The data were analyzed using descriptive statistics.

**Results:**

Patients received an average of 3.47 hippotherapy sessions. During hippotherapy, 66.4% of the predefined goals were reached; cognitive functions and social behavior were the most successfully achieved goals. The most frequently documented patient reaction was had fun (81% of all sessions).

**Conclusion:**

Our study shows that hippotherapy is a feasible and beneficial treatment for neuropediatric patients with severe neurological impairments. The goals of hippotherapy can be much broader than the usually targeted physical functioning and mobility. Hippotherapy might be especially effective in promoting fun and motivation and in addressing patients’ socioemotional needs.

## Introduction

Neuropediatric hospitalization can be a stressful experience for children and adolescents. It can lead to symptoms of anxiety, depression, and distress [1,2]. Besides stressors such as uncertainty, painful procedures, physical limitations, activity restrictions, and decreased independence, inpatients are frequently socially isolated, leading to feelings of loneliness and boredom [2–4]. A review by Melnyk revealed persisting symptoms like sleep problems and negative emotions such as apathy and sadness in hospitalized children [5]. Depressive symptoms frequently accompany physical illness and, notably, hospitalization. A meta-analysis conducted by Walker et al. underlined that adult inpatients show comorbid depression rates ranging from 5 to 34 percent [6]. Additionally, traumatic experiences frequently underlie the reasons for hospitalization, potentially leading to posttraumatic-stress disorders [7]. Addressing these issues is crucial because mental-health conditions correlate with worsened physical symptoms, reduced quality of life, and greater functional impairment [7]. Moreover, psychological burdens are negatively correlated with learning, whereas psychological well-being is positively associated with learning and motivation [8]. In neurorehabilitation the patient’s active participation, learning, and motivation are crucial. Offering therapies that cultivate therapy motivation by enhancing positive emotions and providing relief from the usual clinic setting is therefore a promising approach to enhancing rehabilitation success.

Over the past few decades, hippotherapy has emerged as a physiotherapeutic-treatment option for different populations with impairments of the central nervous system (CNS), autism-spectrum disorders, sensory-processing disorders, and various other neurological and psychological challenges [9,10]. The definition of hippotherapy varies but there is a consensus that it is a form of occupational therapy, physical therapy or speech-language therapy using equine movement as a therapy tool to promote functional outcomes by engaging sensory, neuromotor, and cognitive systems [11,12]. Studies have found evidence that it provides physical benefits for coordination, balance, postural stabilization, gait, sensory processing, and muscle tone [10,13].

While these effects of hippotherapy on physical functioning are well documented, the body of literature regarding the psychological and neuropsychological effects of hippotherapy is small and sometimes even conflicting, with small sample sizes [15–17,19,21], insufficient designs, and a lack of generalization. So far, the effects of hippotherapy have been mainly investigated with regard to depression, grieving, functionality, life habits, well-being, peer acceptance, self-competence, communication, social involvement, and quality of life [13–21]. The interaction with a horse can lead to increased empathy, facilitate therapeutic relationships, and has the potential to increase confidence, social acceptance, self-efficacy, and self-esteem [22]. A narrative review by Rigby summarized the promising effects of equine-assisted services, including hippotherapy, on the human endocrine response [23]. Moreover, Oh et al. found that when receiving hippotherapy, a group of children with attention-deficit/hyperactivity disorder exhibited the same improvements in attention and impulsivity/hyperactivity as a control group receiving pharmacotherapy [24]. Besides this limited research on the psychological and neuropsychological effects of hippotherapy, most of the research on the effects of hippotherapy refers to patients with cerebral palsy [13]. Another limitation of the previous research is that most studies have only included outpatients.

This exploratory study aimed to fill these gaps. Our primary goal was to evaluate the psychological and neuropsychological potential of hippotherapy in pediatric neurorehabilitation by retrospectively analyzing a large dataset of children and adolescents with severe neurological disorders. Our aims were to (1) identify characteristics of patients who had been treated with hippotherapy; (2) identify characteristics of the setting regarding duration, the number of administered sessions, and the predefined neuropsychosocial goals; (3) analyze the documented psychological and neuropsychological reactions of the patients; and (4) analyze the therapists’ perceptions of the extent to which the therapeutic goals were realized.

## Materials and methods

### Participants

We screened data from all the 602 children and adolescents who had been admitted to hippotherapy in a neuropediatric inpatient setting at the Schön Clinic in Vogtareuth, Germany, between June 2001 and October 2020. Data was accessed between 5 November and 27 November 2020. An incomplete record was an exclusion criterion (*n* =  21). The local ethics committee reviewed the study protocols and confirmed that no formal application was required (Bavarian Medical Association, UZ: 2024-1115).

### Study design and procedures

This exploratory study is a retrospective analysis of all available data from all the documented hippotherapy sessions of 581 patients performed between 2001 and 2020. The hospital is a specialist center for pediatric neurology, neurorehabilitation, and epileptology. In this study, we used data recorded on patient-registration forms as well as session-documentation forms to investigate patient characteristics as well as the characteristics and outcomes of the hippotherapy sessions (see S1–S5 Figs).

#### Hippotherapy.

At the Schön Clinic, hippotherapy was prescribed by a multidisciplinary neuropediatric team comprising physicians, physiotherapists, occupational therapists, and nursing staff. They determine the eligibility of patients. Contraindications were insufficient mobility, risk of falling, permanent ventilation, prolonged disorders of consciousness, severe mental disabilities, immunosuppression, or allergies against hay or horsehair. Patients also needed to be able to sit freely for 15–20 minutes, which was assessed by a physiotherapist. Any open wounds needed to be covered with hydrocolloid dressings. If a patient was recommended for hippotherapy, the parents gave written informed consent. After that, a physiotherapist filled out the application form and defined the goals of hippotherapy for the patient. The sessions were conducted by licensed physiotherapists, who had an additional hippotherapy qualification from the German Curatorship for Therapeutic Riding (DKThR). The therapy sessions took place on a riding ground (an open space or a riding hall) 20 minutes from the clinic, where patients were transported by bus or car. The project was financed by the rehabilitation clinic and the nonprofit association Silberstreifen e. V., Germany.

At least two physiotherapists were present during the therapy sessions: one oversaw the handling of the horses, while the other was responsible for the patient. The therapy sessions consisted of three phases: (1) a preparation phase where the patients helped with the cleaning and preparing of the horse; (2) the hippotherapy session, where the patients mounted the horse; and (3) an after-care phase where the patients helped with removing the riding pad, washing, and feeding the horse. Phases 1 and 3 provided opportunities for the patients to engage in cognitively stimulating tasks like action-planning, caretaking, and interacting with the horse, the therapists, and other patients. Furthermore, motor functions and physical skills were trained by, for example, handling a horse brush.

In phase 2, the patients accessed the sandy riding ground or the riding hall (depending on the weather conditions). According to their mobility, they do so with or without the help of therapists or family members, walking aids, or a wheelchair. The horse and the therapist in charge of the horse were waiting next to a specially constructed mobile staircase that the patients could use to mount the horse. The mounting aid was wheelchair accessible. The transition to horseback was performed with external aid determined by the patient’s mobility. The patients were required to wear a helmet unless there was a physiotherapeutic contraindication and were secured with a trunk harness that the therapist held on to. In case of emergency, the therapist could pull the patient to their side and prevent them from falling off the horse. Between 2001 and 2020, no such incidents occurred. Following checks of the proper safety procedures, the therapist in charge of the horse would position themselves behind the horse and issue commands to the horse for walking and stopping. The therapist in charge of the patient walked alongside the patient where they could interact and give tactile impulses to help the patient center themselves and maintain their balance if necessary.

During the hippotherapy sessions (phase 2), patients were often given tasks like to lie on the horse’s back, to lift their arms, or to touch the horse’s tail or mane. The direction of the track changed during the session to avoid one-sided strain for both horse and rider. After an average period of 20 minutes, the patient demounted the horse. The patient could then either wait until the next patient had finished mounting or could help with unsaddling and caring for the horse (phase 3). At the end of each session, the therapists discussed the observations and behavior of the patient and filled in the session-documentation form.

All patients received hippotherapy in addition to other treatments and therapies in the hospital.

#### Horses.

A total of four horses (Black Forest gelding; Icelandic gelding; Icelandic mare; Holstein mare) were involved in the hippotherapy sessions between 2001 and 2020. All were trained by an equestrian and physiotherapist. The horses were chosen according to their temperament and character. Each horse worked two days a week; on each day they completed a maximum of five sessions of approximately 20 minutes each. It was ensured that the horses received enough free time before and after each therapy session. To prepare and train the horses for the unbalanced weight exposure during hippotherapy, they were mounted for correction twice a week by a classic dressage rider and also taken out in the countryside by one of the horsekeepers to keep them balanced and well-trained. The horses lived in a species-appropriate active stable with the ability to move freely within a paddock and a meadow, to interact with the rest of the herd, and to access fresh water and hay ad libitum. For the hippotherapy sessions, the horses were bridled with a bit and saddled with a vaulting girth and pad. The therapist steered the horse with the aid of long reins, a riding crop, and voice commands from a position behind the horse.

### Outcomes

#### Patient characteristics.

For every patient receiving hippotherapy, a registration form was filled out. Between 2001 and 2020, two different types of forms were used (see S1–S2 Figs). The old registration form was used between 2001 and 2011; it collected data about the patient’s age, diagnoses, and contraindications, as well as qualitative data about the problem statement and goals of the hippotherapy. The new registration form was in use from 2012 until the end of data collection in 2020; it contained information about the patient’s age, diagnoses, mobility, communication ability, noteworthy special characteristics, and contraindications, as well as other clinic-related data and standardized goals for physiotherapy and occupational therapy. The form provided a set of predefined therapeutic goals that could be chosen by ticking a box; only eight of the choices were relevant for this study since we focused on neuropsychosocial goals.

To analyze the level of communication skills, we split the category “communication” into “speaking ability” and “speech comprehension”. Within the category of speaking ability were six subcategories, while speech comprehension consisted of four subcategories. Patients were assigned to more than one subcategory if appropriate.

The patients’ diagnoses were assigned to the following four etiological categories for analysis, each consisting of subcategories: 1. innate or perinatal injury of the CNS; 2. acquired injury of the CNS; 3. muscular disorder; and 4. miscellaneous. Patients were assigned to more than one subcategory if appropriate.

The analysis of the patients’ mobility was made according to the Gross Motor Functions Classification System (GMFCS) [25]. The GMFCS is a classification tool that was developed for children with cerebral palsy, but it has also been validated for children with Down syndrome and for children suffering from a traumatic brain injury. Patients can be assigned to five different GMFCS levels on an ordinal scale. Patients on level 5 of the GMFCS have the most difficulties with mobility, while patients on level 1 of the GMFCS exhibit the fewest difficulties (see S6 Table).

#### Session documentation.

Every hippotherapy session was documented using a semistructured paper-and-pencil form developed for quality assurance. The therapists completed the form together after each session. Over the years, three different types of session-documentation forms were used. One form was used between 2001 and 2011 (see S3 Fig), one form only in 2012 (see S4 Fig), and the newest form was used between 2012 and 2020 (see S5 Fig). The session-documentation forms included the date, session number, session length, and the involved therapists. For this study, we extracted qualitative data about the patient’s behavior, cognition, and daily condition from these forms, as well as the number of attended hippotherapy sessions.

#### Predefined therapeutic goals.

The information on the patients’ registration forms (see S1 and S2 Figs) was used to identify each participant’s individual neuropsychosocial goals for therapy. All the text was transcribed and then categorized by performing a shortened version of the content analysis outlined by Erlingsson and Brysiewicz [26] in line with a previous study investigating dog-assisted therapy in the same hospital [27]. With this method, all the nonrelevant passages are deleted in the first step, while the core meaning, meaning units, and codes remain. As a next step, the codes and meaning units are clustered into categories. Two independent examiners (ML and EM) separately reviewed the categorization process. Discrepancies in the coding and categorization were discussed and resolved by consensus. In the new version of the registration form (see S2 Fig), therapists indicated therapeutic goals by ticking a box. These data were therefore more structured and we used the categories provided. We used the categories that we obtained by content analysis or by using the existing categories on the forms to obtain descriptive statistics by counting how many goals were mentioned for each patient.

#### Goal realization.

We employed the two categories “daily form/behavior” and “cognition/psyche” from the session-documentation forms used between 2012 and 2022 to evaluate the extent to which the predefined neuropsychosocial goals for therapy were achieved during the hippotherapy sessions. A shortened version of the content analysis outlined by Erlingson and Brysiewicz was performed to develop categories [26]. Afterward, categories that consisted of the opposite behavior were added to each of the identified categories to ensure objective results. For example, “does not help” was the added alternative to “helps,” “unmotivated” was the added alternative to “motivated,” and “stressed” was the added alternative to “relaxed”.

**Fig 1:**
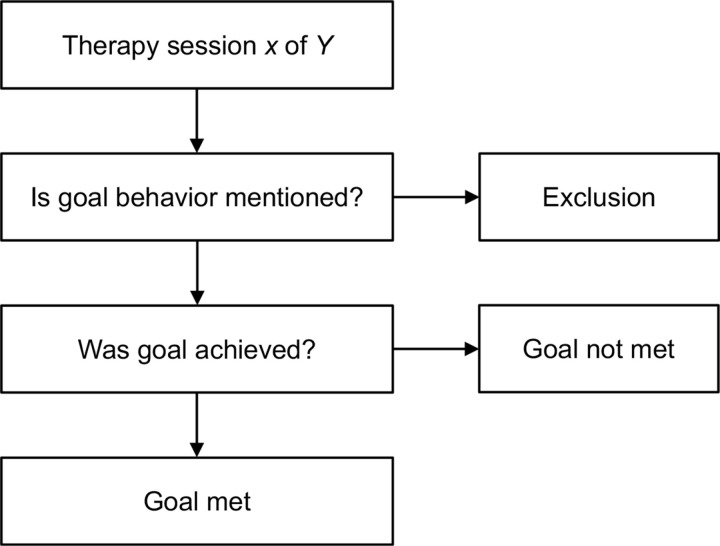
Process of evaluating whether the predefined therapeutic goal was met in an individual for a certain therapy session.

The extent to which the predefined goals were met was evaluated for each patient individually as shown in Fig 1. Then the ratio of the sessions that contained information relevant for evaluating goal achievement *n*_fulfilled_ to the total number of sessions *n*_included_ was calculated as *n*_fulfilled_/*n*_included_. For example, if participant *Y* was given the goal of engaging in group interactions, the process was the following: In 4 out of 13 sessions, the participant interacted with other patients, but in 6 out of 13 sessions, the participant did not engage in group interactions, and in three sessions, group interaction was not mentioned. The sessions with missing data were removed from the total, leaving 10 sessions total. Out of those 10, the patient interacted with other patients 4 times and did not interact with other patients 6 times. Accordingly, the goal was only reached in 40% of the therapy sessions and therefore declared as not met with a reported exclusion of 3 sessions.

#### Further reactions.

To explore the full range of behaviors observed during hippotherapy, the number and percentage of therapy sessions in which certain behaviors were observed were noted by the therapists, regardless of whether a therapeutic objective was defined or not. To analyze these observed behaviors, the frequency of all identified categories from the session-documentation form was considered, and all sessions from all patients who had at least one behavioral observation were included.

### Statistical analyses

**Fig 2:**
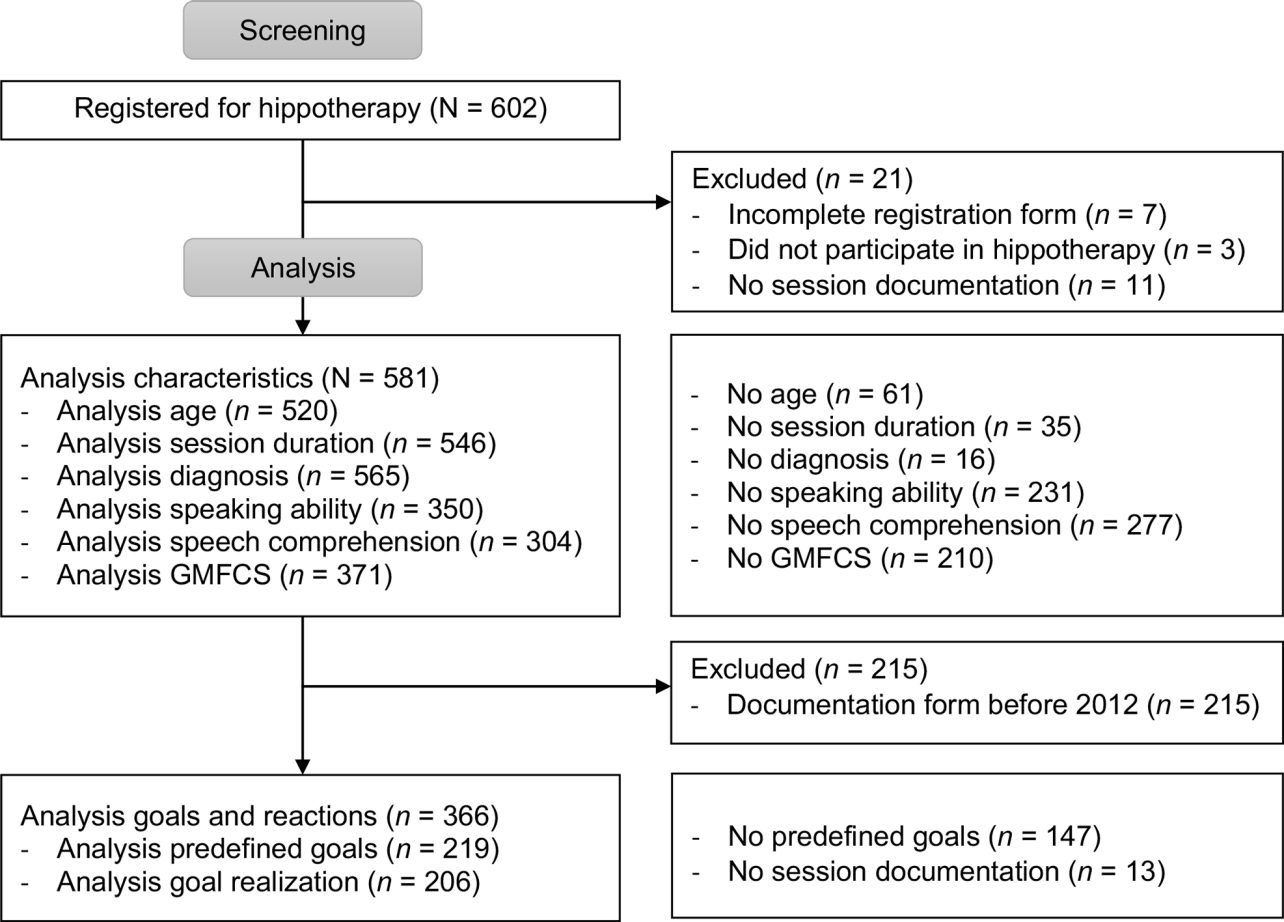
Flow chart.

All data were matched with the patient’s health records to gather information on sex, date of birth, and date of admission, then anonymized and digitally transcribed. Anonymization was carried out by the hospital staff by assigning a number to each patient. Researchers not affiliated with the hospital received only anonymized data for analysis. All handwritten comments on the admission and course documentation sheets were transcribed. After having transcribed all data, we screened data for inclusion/exclusion criteria. We used the available data from June 2001 to October 2020; they had been recorded for quality assurance. The data from 602 patients were screened. Eighteen patients had two registration forms, and one patient had three (these children had several periods at the clinic), resulting in 622 registration forms for 602 patients. Seven patients had incomplete registration forms, three patients did not start with hippotherapy, and 11 did not have a filled-out session-documentation form. These 21 patients were excluded from the data analysis. Thus, a total of 581 patients met the inclusion criteria, leading to a dataset of 2031 analyzed sessions (see Fig 2).

The analyses of age, session duration, diagnosis, communication skills, and mobility level were based on different numbers of patients due to missing data (see Fig 2). Sixty-one registration forms had missing data about the patient’s ages, so they were excluded from the calculation of the average age. Thirty-five sessions were excluded from the analysis of the session duration because it was not noted on the documentation form. Gender was analyzed for all 581 patients. Sixteen of the 581 registration forms did not contain information about diagnoses, 231 of them did not have any data about speaking ability, and 277 forms did not have any information about speech comprehension. Forty-three patients already had a GMFCS score from a physiotherapist, and 328 patients were categorized to a GMFCS score based on the data in the mobility section of the form by the study team. For 210 patients, no information about mobility was available.

The analysis of reactions and goal achievement included the 366 patients whose sessions were documented with the session-documentation form that was used from 2012 to 2020. Previous session-documentation forms were excluded from the reaction and goal analysis as they were too different to compare.

Descriptive analyses were performed with R version 4.1.2 in R Studio version 2021.09.2 [28].

## Results

### Participant characteristics

**Fig 3:**
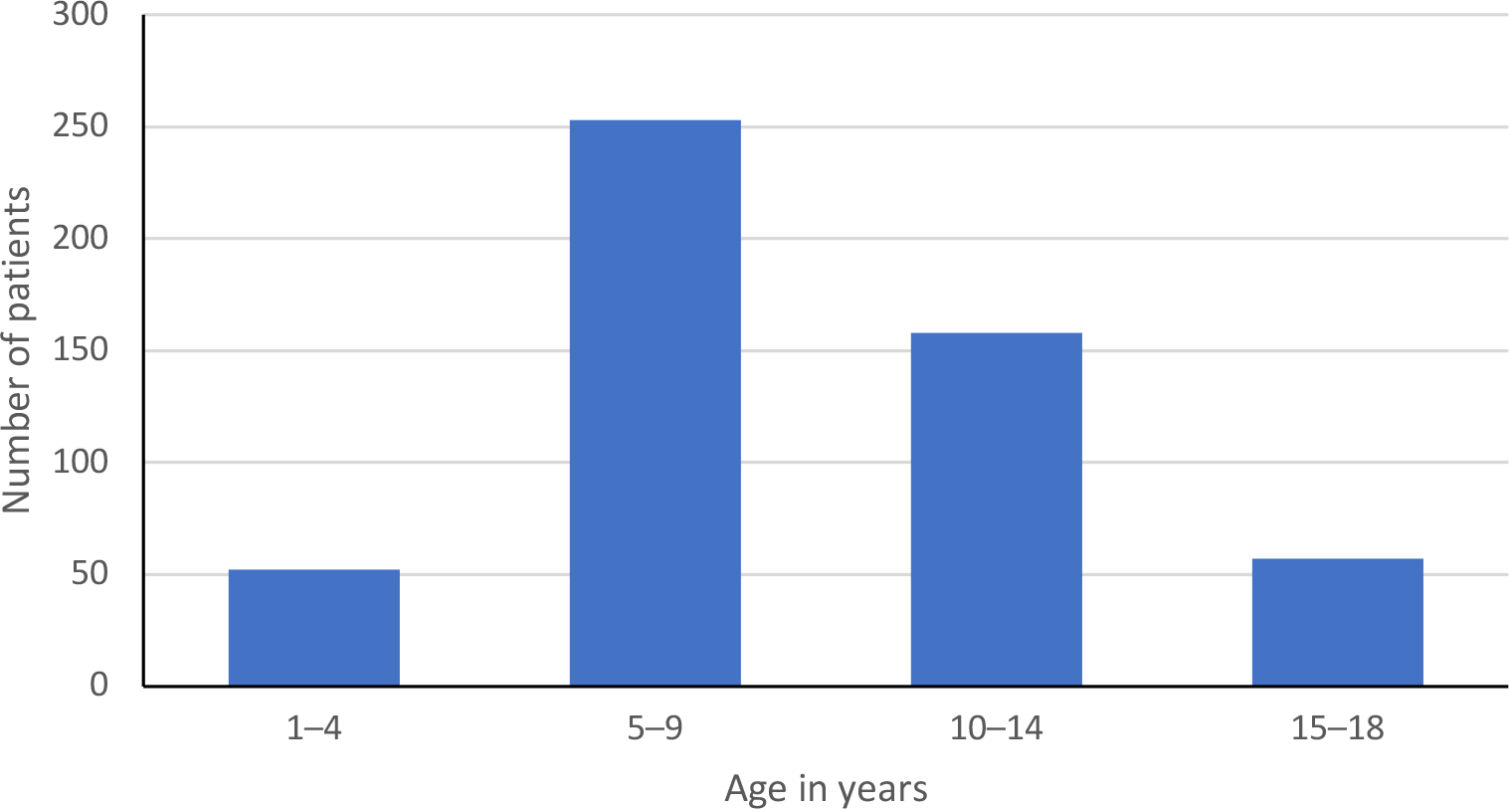
Age of patients in years at the time of admission to their first hippotherapy session (*n* =  520 patients).

**Fig 4:**
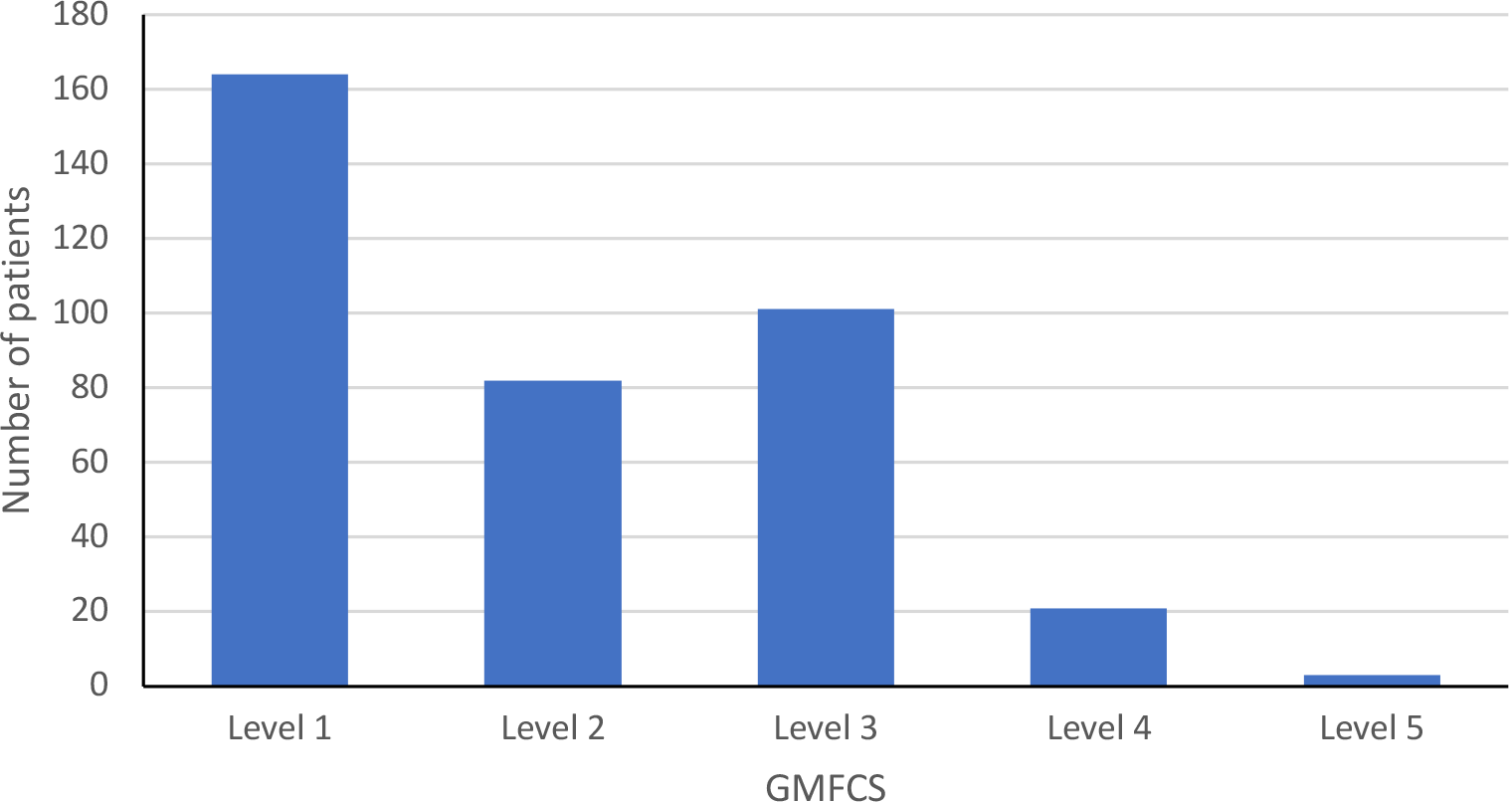
Patients’ GMFCS levels at the time of admission to their first hippotherapy session (*n* =  371 patients).

In total, there were 581 children and adolescents (305 female, 276 male) who had received hippotherapy at least once and for whom there was at least one complete registration form and one session-documentation form. The mean age was 9.01 years (*SD* =  3.88), ranging from 1 to 18 years (see Fig 3). Of all the patients, 55% had an innate injury of the CNS, while 68% had an acquired CNS impairment (see Table 1). Patients’ communication skills and GMFCS levels at the time of their admission to their first hippotherapy session are presented in Table 2 and Fig 4.

**Table 1:** Diagnoses (*n* =  565 patients, multiple answers possible).

Etiological category	Number of patients	%
*Innate or perinatal injury of CNS*	*313*	*55.40*
Genetic disorders	58	10.27
Premature infant	34	6.02
Cerebral palsy	221	39.12
Hemiparesis	119	21.06
Diparesis	49	8.67
Tetraparesis	22	3.89
Not further specified	31	5.49
*Acquired injury of CNS*	*385*	*68.14*
Brain/cerebellum injury	295	52.21
Inflammatory disease	57	10.09
Traumatic injury	115	20.35
Vascular disease	70	12.39
Tumor	35	6.19
Other acquired brain injury	18	3.19
Spinal cord injury	26	4.60
Injury of the peripheral nervous system	12	2.12
Other acquired injury	52	9.20
*Muscular disease*	*7*	*1.24*
*Miscellaneous*	*218*	*38.58*
Epilepsy	104	18.41
Pediatric orthopedics	29	5.13

**Table 2:** Communication skills (*n* =  350/304 patients, multiple answers possible).

Communication category	Number of patients	%
*Speaking ability*	*350*	
Speaks without any problem	197	56.29
Verbally difficult to understand	53	15.14
Only single words/uses gestures	51	14.57
Slowed speech	31	8.86
Cannot speak	6	1.71
Speaks a foreign language	36	10.29
*Speech comprehension*	*304*	
Understands everything	242	79.61
Understands little	28	9.21
Understands nothing	2	0.66
Understands a foreign language	32	10.53

### Hippotherapy characteristics

**Fig 5:**
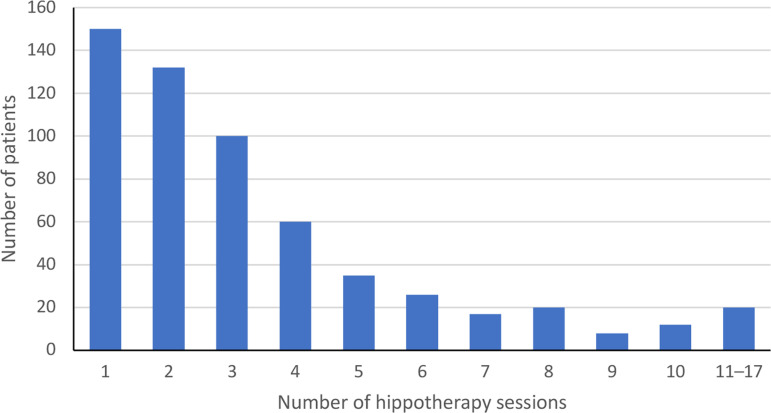
Number of hippotherapy sessions per patient (*N* =  581 patients).

In total, 2031 hippotherapy sessions were conducted during the evaluation period between June 2001 and October 2020. On average, patients received 3.47 sessions (*SD* =  2.85), with a range between 1 and 17 sessions (see Fig 5). The duration of a hippotherapy session was on average 20.62 minutes (*SD* =  4.47) and ranged from 5 to 30 minutes.

### Predefined therapy goals

The predefined goals for hippotherapy targeted emotional, psychological, neuropsychological, and social functioning and were divided into eight different functional areas that were preset by the rehabilitation team on the registration form.

Frequency analysis revealed that every participant had a median of two and between one and eight defined neuropsychosocial goals for therapy before starting with hippotherapy (*M* =  3.3, *SD* =  1.7), resulting in 682 predefined therapeutic goals (see S7 Table). The most frequently assigned goals aimed at facilitating group interaction (*n* =  143), concentration (*n* =  115), and interaction with humans (*n* =  110), followed by action-planning/problem-solving (*n* =  99), reduced anxiety (*n* =  71), and respecting the rules (*n* =  57). Memory/retentiveness (*n* =  51) and showing consideration (*n* =  36) were the least often predefined therapeutic goals.

### Goal realization

The 366 patients with session-documentation forms had a total of 1142 documented sessions; of those 366 patients, 206 had both predefined goals and session-documentation forms. The 629 sessions of those 206 patients were used for the goal-realization analysis. We further excluded sessions that did not explicitly document positive or negative behavior that could be used to evaluate the extent of goal attainment.

Out of 682 predefined neuropsychosocial goals for therapy within the sample, 66.4% were met. The most successfully accomplished therapeutic goals included cognitive functions like action-planning/problem-solving (88.7%), memory/retentiveness (80%), and social behavior such as interacting with the group (81%) or with other humans (84.6%). An almost equal ratio of positive and negative outcomes was found in reduced anxiety (48.1%). The lowest achievement ratios were found in socially relevant functions like respecting the rules (42.8%) and concentrating (39.2%). For a detailed overview of the goal achievements and exclusions, see S8 Table.

The validity of the goal-achievement evaluation is related to the number of sessions that contained information relevant for evaluating goal achievement in relation to the total number of sessions. The most data-rich information was obtained for interacting with humans (47.0% of all sessions, *n*_*included*_ =  157 sessions) and action-planning/problem-solving (53.3% of all sessions, *n*_*included*_ =  152 sessions). The ratio of sessions that included relevant information (defined as *n*_included_/*n*_total_) was lower than 30% for concentration (29.7%, *n*_*included*_ =  109), reduced anxiety (24.5%, *n*_*included*_ =  55), and respecting the rules (9.6%, *n*_*included*_ =  16). For group interaction (4%, *n*_*included*_ =  18), memory/retentiveness (2.9%, *n*_*included*_ =  5), and consideration (4.4%, *n*_*included*_ =  4), the ratio was very low (see S9 Fig).

### Further reactions of the patients

The psychological and neuropsychological reactions of the 366 patients during 1142 sessions were categorized into 28 categories. Of those, 14 were actual documented reactions formulated positively as the desirable behavior. The other 14 categories were added and consisted of the corresponding inverse reactions as described in the methods. We identified more reactions to hippotherapy sessions than the eight predefined neuropsychosocial goals defined on the registration forms, but those goals were all among the identified categories (see Table 3).

**Table 3:** Patients’ reactions. Eight categories were defined by the hospital on the registration form; six were added post hoc from the content analysis of the registration forms. The 14 inverse categories were also added post hoc.

	Reaction category	Inverse reaction category
Predefined neuropsychosocial goals by the hospital	Good action-planning/problem-solving	Bad action-planning/problem-solving
	Good memory/retentiveness	Bad memory/retentiveness
	Concentrated	Not concentrated/distracted
	Reduced anxiety	Anxiety
	Group interaction	No group interaction
	Interaction with humans	No interaction with humans
	Respected the rules	Did not respect the rules
	Showed consideration	Did not show consideration
Additional identified reactions	Had fun	Sadness (crying)
	Helpful	Not helpful
	Patient/relaxed	Impatient/stressed
	Without family	With family
	Motivated	Unmotivated
	Interaction with the horse	No interaction with the horse

**Fig 6:**
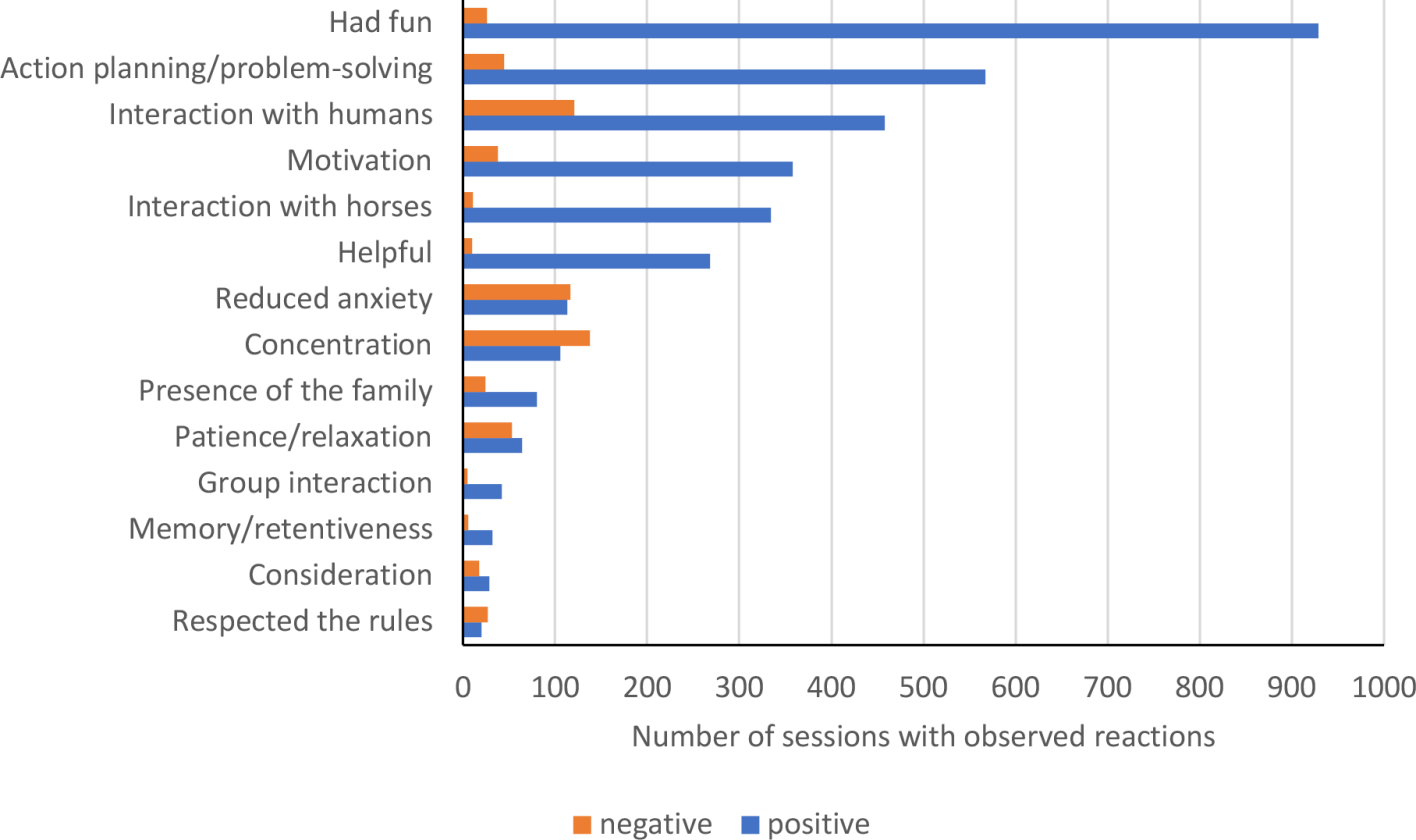
Documented reactions in patients during hippotherapy sessions (*n*_total_ =  1142 documented sessions).

The most frequently documented reaction of the patients was had fun (in 81.1% of all sessions), and an explicit description of did not have fun during the session (2.2%) was very rare. This was followed by good action-planning and problem-solving (49.5%) and actively interacting with other humans (40%) (see Fig 6 and S10 Table). In 31.2% of the sessions, the patients were motivated, while unmotivated patients were only reported in 3.3% of the sessions. It was more often the case that patients were documented as unconcentrated (12%) than concentrated (9.2%). The least documented reactions were patience/relaxation (5.6%), group-interaction (3.7%), memory/retentiveness (2.7%), consideration (2.5%), and respected the rules (1.7%).

## Discussion

We retrospectively analyzed data from children and adolescents with severe neurological disorders who had received hippotherapy in inpatient neurorehabilitation to identify characteristics of the treated patients and to gain insight into the characteristics of the sessions, the targeted neuropsychosocial goals, the patients’ psychological and neuropsychological reactions, and goal realization. We found that the hippotherapy sessions were attended by children and adolescents between the ages of 1 and 18, the majority being between 5 and 9 years old. The patients’ GMFCS levels ranged from 1 to 5, and the majority were between 1 and 3, meaning that most of the patients were at least mobile with assistive mobility devices or wheelchairs. Of all the patients, 40% had difficulties speaking or could not speak at all, while 80% understood speech fully, and only 10% had difficulties or did not understand spoken language. This indicates that while mobility and speech comprehension are important for patients in hippotherapy, it can still be a viable treatment even without those skills.

Around 68% of the patients were suffering from an acquired injury of the CNS, and 55% had an innate or perinatal disorder, meaning that some patients were affected by both. Of the patients with innate or perinatal injuries of the CNS, 71% were suffering from cerebral palsy. This reflects the large amount of literature investigating how hippotherapy affects patients with cerebral palsy [13,20,29,30]. Most of the disorders observed in the current patient sample were related to the brain, traumatic brain injuries being the primary cause. Many patients receiving hippotherapy had been diagnosed with epilepsy. Before admission, the interdisciplinary clinic team assessed the eligibility of each patient, ensuring that only patients with a low risk of sudden seizures were permitted to participate in hippotherapy.

This explorative study documents that hippotherapy is feasible with very young and severely affected patients as long as a thorough screening process and proper protocols are in place. From 2001 to 2020, there were not any registered incidents where a patient or a horse was harmed. One session had to be stopped early because a patient had an asthma attack after arriving at the riding ground. No other critical incidents or adverse effects were recorded.

The average length of a hippotherapy session was 21 minutes, with a range between 5 and 30 minutes, which shows that flexibility is important as the duration needs to be adapted to the patient’s needs. Compared with other hippotherapy studies, the session length was a bit shorter than the average of 38 minutes reported in a systematic mapping review that included 78 studies on hippotherapy [13]. In our study, patients received three hippotherapy sessions on average, with a range between 1 and 17 sessions, whereas the mapping review revealed an average of 17.8 sessions with a range between 1 and 104 sessions [13].

In this study, the patients were involved not only in mounted activities while on the horses’ back but also in human–horse interactions on the ground before and after as an important part of the sessions. Thus, as it was documented at this clinic, hippotherapy not only encompassed equine movement guided by physiotherapists but also elements from the broader spectrum of equine-assisted therapy [12,13]. Wood and colleagues [13] found that this is not common and usually only reported in studies that address psychosocial matters [13–21].

The predefined goals targeted emotional, psychological, neuropsychological, and social functioning, the most frequently defined goals targeting social skills (group interaction and interaction with humans). Other frequently set goals concerned cognitive abilities. These results indicate that the predefined goals in this sample were much broader compared to the most prominent outcomes of hippotherapy, which are physical functioning and mobility [13,20].

The documentation of the involved therapists after each session revealed that the most frequently realized goals were related to cognitive functions like action-planning/problem-solving and memory/retentiveness or to social functioning such as interacting with the group or with other humans. Goals like respecting the rules and concentration had the lowest achievement ratios. Overall, 66% of the predefined therapeutic goals were met in the hippotherapy sessions. Moreover, the documentation of the therapists revealed a broad range of additional patient reactions beyond the predefined neuropsychosocial goals for therapy. These additional observations were related to having fun, being helpful, being patient/relaxed, being without the family, motivation, and interaction with the horse. The most frequently documented reaction of the patients during hippotherapy sessions was had fun. Moreover, the analysis revealed that the patients were motivated and engaged in cognitively stimulating tasks such as action-planning and problem-solving, and that they displayed social behavior by actively interacting with other humans and the therapy horses.

In previous studies investigating the effects of hippotherapy, the large majority of reported outcomes have suggested improvements in physical functioning and mobility mostly in movement-related functions including postural control and balance, coordination, gait, and in mobility in daily life [13,20]. Other commonly reported positive outcomes have been improvements in daily-life activities, especially self-care [13]. The psychosocial measures used in hippotherapy studies have targeted depression, happiness, general well-being, peer acceptance, self-worth, communication, social involvement, and quality of life [13–18]. In contrast to hippotherapy, studies investigating equine-assisted therapies in general—and with a broader range of patients—have more often included psychosocial measures such as social skills, communication, activity and participation, self-care, behavioral or psychological symptoms, and quality of life [20,31–33]. They have produced high-quality evidence that equine-assisted therapy enhances quality of life, medium-quality evidence for activity and participation, and low quality for communication [20]. Previous research on broader services such as animal-assisted therapy has indicated that interacting with animals can lead to enhanced social behavior, interaction, and communication in patients in neurorehabilitation [27,34,35] and suggests that animals can lead to better interaction and an improved therapeutic alliance between the therapist and the patient [36–38].

Our results outline the potential of hippotherapy to foster socioemotional functioning beside the well-documented effects on physical functioning and mobility. This is especially relevant since hospitalization often leads to social isolation [2,4]. Moreover, the results indicate that hippotherapy can be a valid approach for enhancing fun and motivation. This is in line with a review that found that 22% of studies suggest that hippotherapy favorably affects motivation and confidence, which is sometimes used as an explanation and underlying mechanism for the effects of hippotherapy [13]. Shurtleff and colleagues even hypothesize that the motivation and fun derived from riding a horse may be more important than the horse’s rhythmic movements [39]. Moreover, fun and motivation are crucial when it comes to the general effectiveness of therapy [40].

It needs to be noted that the documentation forms in the present study were not designed to collect the psychological and neuropsychological effects of hippotherapy. Thus, children’s positive and negative reactions were only documented by the therapists if there was a specific problem such as anxiety. This means that the documented effects represent only a fraction of the potential effects hippotherapy has on mental health.

Interestingly, we found an almost equal ratio of positive and negative outcomes in the reduction of anxiety, and patients were more often documented as being unconcentrated than concentrated. This is contrary to a study that found that therapeutic horseback riding led to improved directed attention and decreased inattention and distractibility in children with autism-spectrum disorders [41]. It is important to also look at the unfavorable effects of hippotherapy to make an informed decision about the indication of this form of therapy for individual patients, as unfavorable effects have also been reported by Wood and colleagues in their mapping review [13]. The review revealed more negative than positive reported outcomes in the domain of communication and an equal number of negative and positive reported outcomes in psychosocial functioning, which is contrary to our findings. We found that the less frequently a behavior was documented, the more similar were the number of times that positive and negative behavior were documented. A possible explanation is that the negative behaviors in these categories stood out as very disturbing and were therefore documented, whereas in the absence of disturbing behaviors, the positive behavior passed by unnoticed. This highlights the importance of systematic observation and the use of standardized measurements to identify the potential and the whole spectrum of the possible effects of hippotherapy.

### Strengths, limitations, and future directions

The results are based on the retrospective analysis of subjective semistandardized ratings by the involved therapists at the end of each hippotherapy session and should therefore be interpreted with caution. Since the documentation sheets were designed for practical use and quality assurance rather than for research, the risk of bias by the therapists who documented the sessions might be lower, but the possibility of bias must be borne in mind. Also, the documentation forms changed over the time period in consideration, which limited the comparability of the present dataset. Since the documentation forms were not intended to measure goal achievement, there was a lot of missing data, which reduced the validity of the goal-achievement evaluation. Also, behaviors that did not stand out might not have been noted, which could have resulted in unrepresentative documentation. A lot of effects might have been missed, as reactions were often only noted for patients who had a specific psychological or neuropsychological problem. Our findings are based on qualitative data and the nature of this study is exploratory. Qualitative data can be very valuable in identifying new and unexpected aspects, providing a deeper understanding of a topic and generating new hypotheses. However, our hypotheses should now be investigated with study designs that can test them, using quantitative data. In future studies, patient reactions and goal realization should be measured by independent raters using standardized tools such as reliable assessments, video coding, or physiological variables. We suggest to combine qualitative with quantitative measures in a mixed-method design. Since there was no control group, the effects found in this study cannot be causally attributed to hippotherapy. It is only possible to derive hypotheses about potential effects. These hypotheses should be tested in controlled investigations in the future. Moreover, future research should identify the effects of specific components of such a complex intervention as hippotherapy. For example, it would be important to distinguish between the core component of movement on a horse from components like interacting with and building a relationship with an animal, which is also part of other equine-assisted or even other animal-assisted treatments. Wood and colleagues even suggest that the term hippotherapy should not be used any longer since it has not been defined clearly enough [12].

The clear strengths of the present study are the large dataset and the fact that the data were not collected for a specific, narrow research question. This makes it possible to gain a broad insight into a practice that is quite common in neurorehabilitation but often only investigated under the aspect of enhancing physiological functioning. This study is also one of the only studies with a qualitative approach, as most studies have quantitatively measured the outcomes of hippotherapy [13]. Future studies should more broadly explore the full potential of hippotherapy, investigate which children and adolescents in inpatient neurorehabilitation it can be beneficial for, and determine the circumstances when the involvement of a different animal (e.g., a dog compared to a horse) might be the best approach for achieving the defined rehabilitation goals.

### Conclusion

This study shows that hippotherapy is feasible, can be beneficial for pediatric patients with diverse neurological impairments, and has the potential to address socioemotional functioning. The goals of hippotherapy can be much broader than physical functioning and mobility that are usually targeted. Our results suggest that involving a horse in physiotherapy might be especially effective in fostering fun and motivation and in addressing a patient’s socioemotional needs. Hippotherapy can therefore be a valuable approach to holistic inpatient neurorehabilitation for children and adolescents with severe neurological impairments.

## Supporting information

S1 Fig Old registration form. (TIF)

S2 Fig New registration form. (TIF)

S3 Fig Session documentation form used 2001–2011. (TIF)

S4 Fig Session documentation form used 2012. (TIF)

S5 Fig Session documentation form 2012–2020. (TIF)

S6 Table Gross Motor Function Classification System (GMFCS). (Cerebral Palsy Alliance Research Foundation, 2024, retrieved from: https://cparf.org/what-is-cerebral-palsy/severity-of-cerebral-palsy/gross-motor-function-classification-system-gmfcs/, based on Palisano et al., 1997 [25].
(DOCX)

S7 Table Frequency of assigned predefined neuropsychosocial therapy goals. (DOCX)

S8 Table Ratio of achieved predefined neuropsychosocial goals in hippotherapy sessions. The total number of sessions refers to the sessions by all the patients who had this goal. Excluded sessions report the number of sessions excluded due to the lack of information on the goal. The achieved column shows the ratio of positive and negative behavior in %.
(DOCX)

S9 Fig Ratio of included and total sessions for each reaction (*n*_included_/*n*_total_). (TIF)

S10 Table Documented reactions of patients during hippotherapy sessions. Presence of the family is grouped under positive behavior. Percentages are calculated attains the *n*_total sessions_ = 1142.
(DOCX)
