# Supplementary figures and images for: Psychological aspects of hippotherapy for children with severe neurological impairment: An exploratory study
Source: PLoS One. 2025 Apr 8;20(4):e0320238. doi: 10.1371/journal.pone.0320238 (PMC11978075; doi:10.1371/journal.pone.0320238)

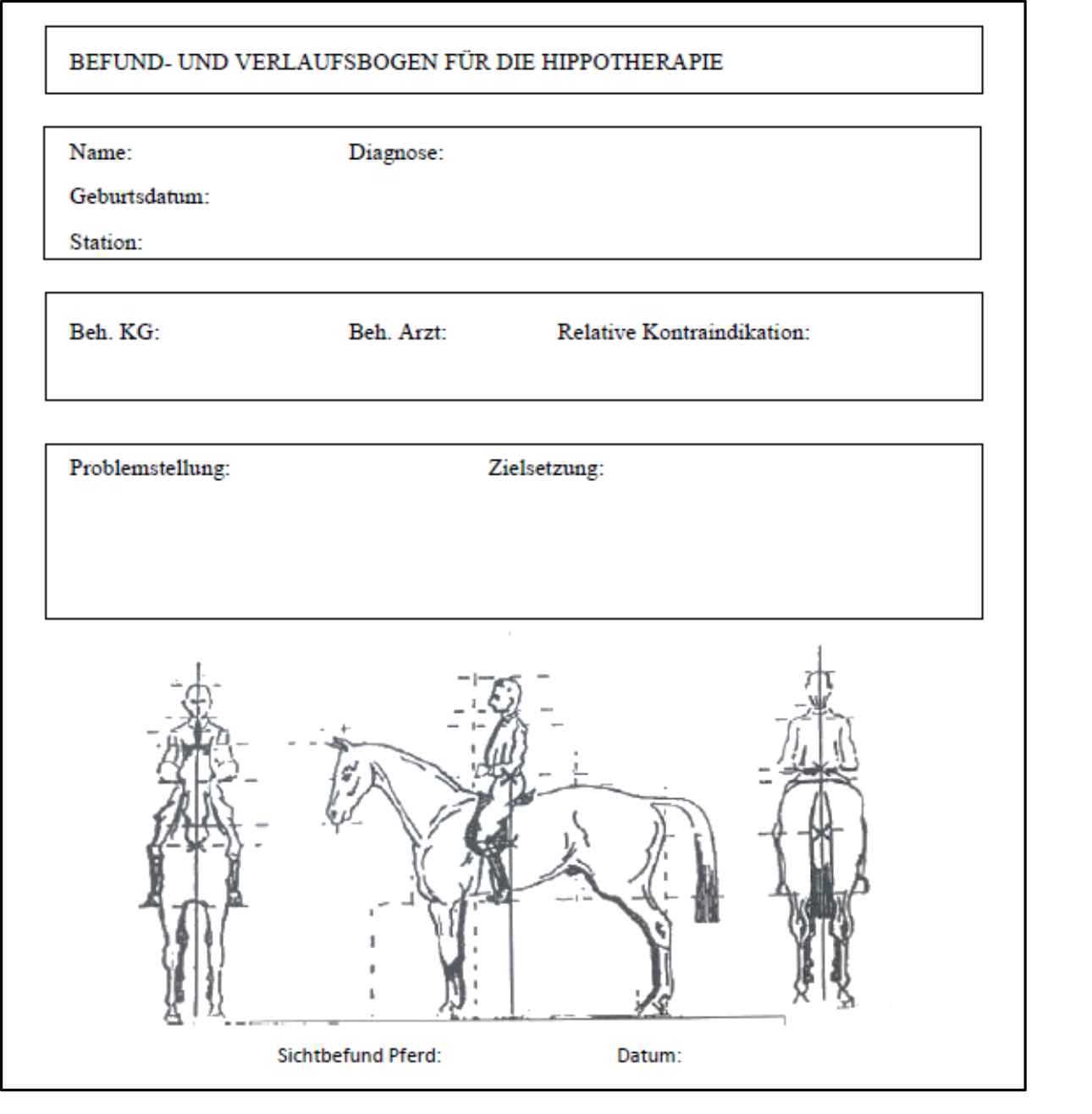

Supplement: S1 Fig — (TIF) [file pone.0320238.s001.tif]

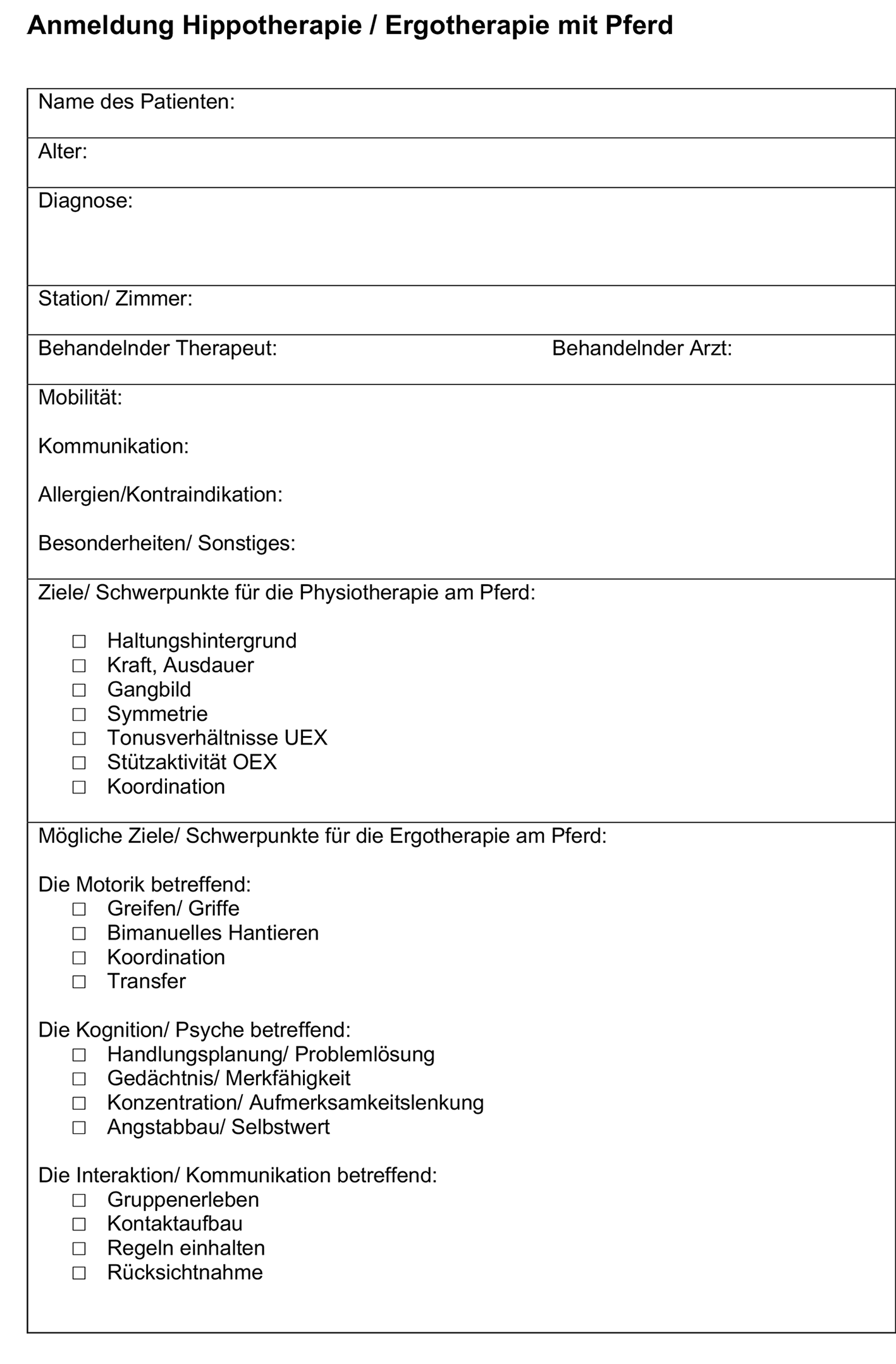

Supplement: S2 Fig — (TIF) [file pone.0320238.s002.tif]

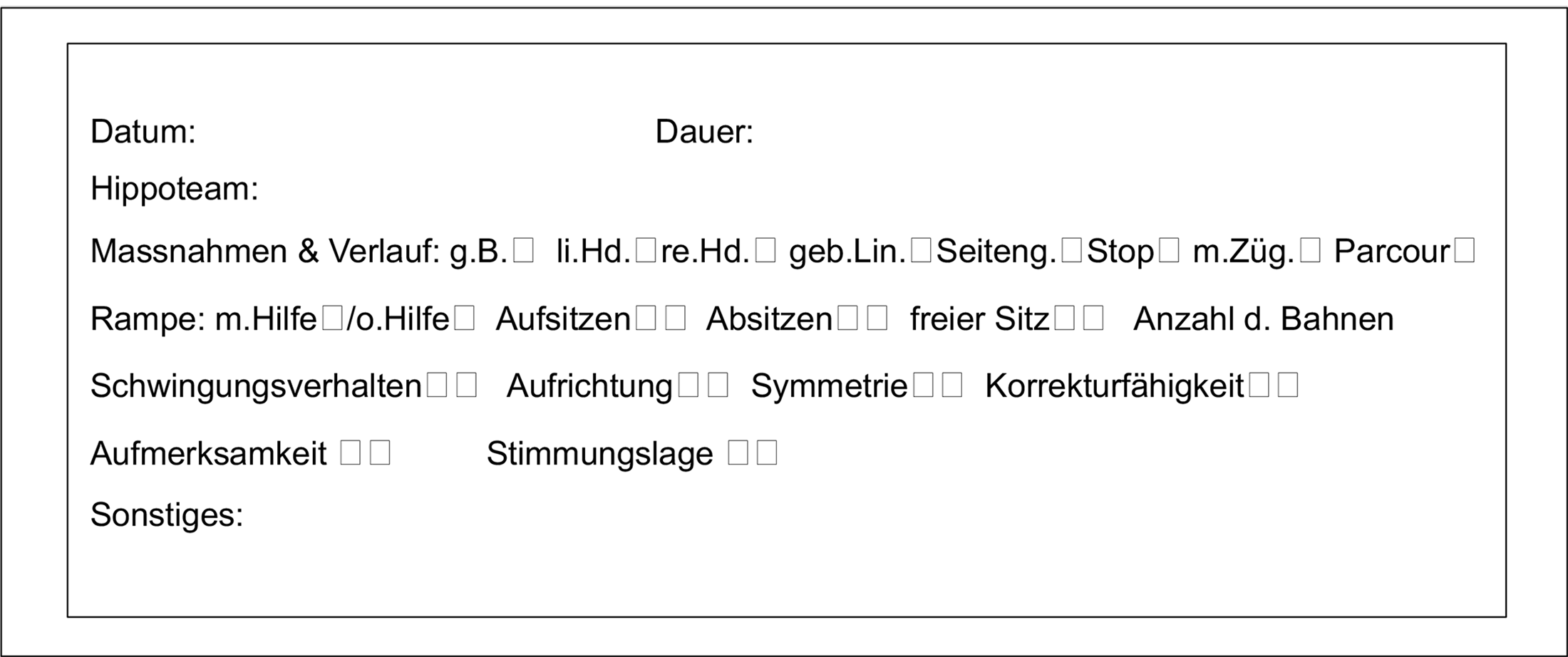

Supplement: S3 Fig — (TIF) [file pone.0320238.s003.tif]

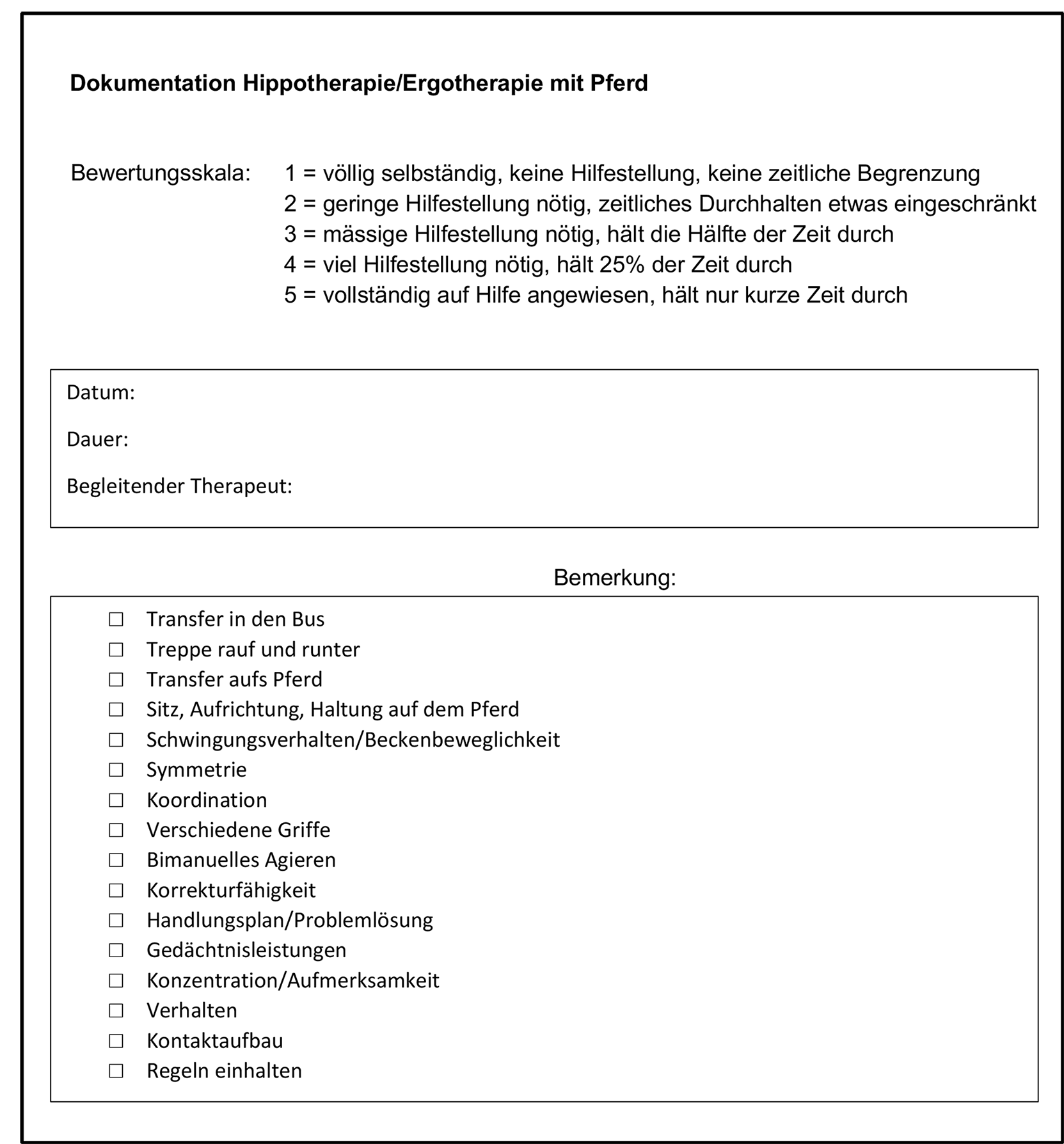

Supplement: S4 Fig — (TIF) [file pone.0320238.s004.tif]

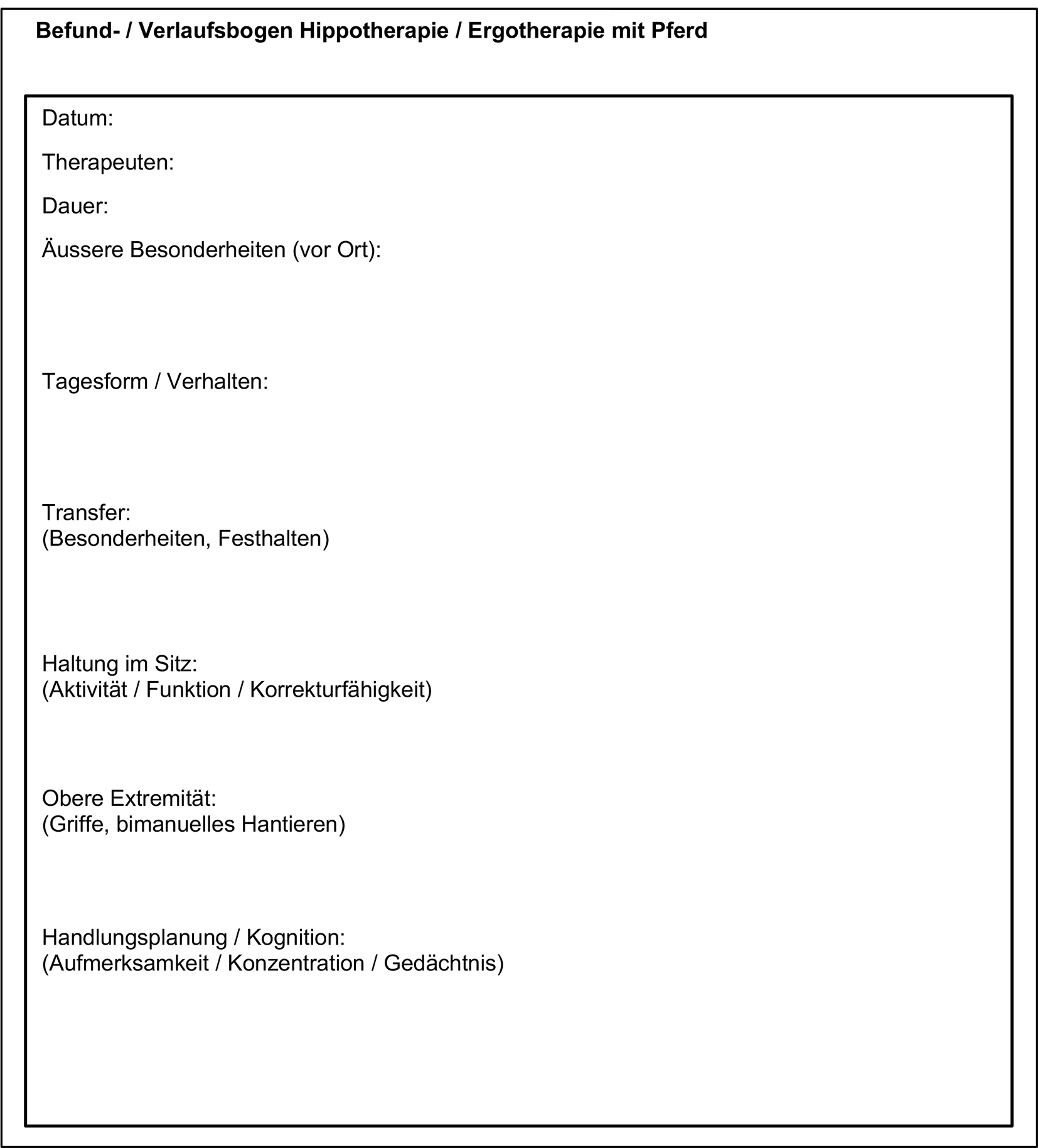

Supplement: S5 Fig — (TIF) [file pone.0320238.s005.tif]

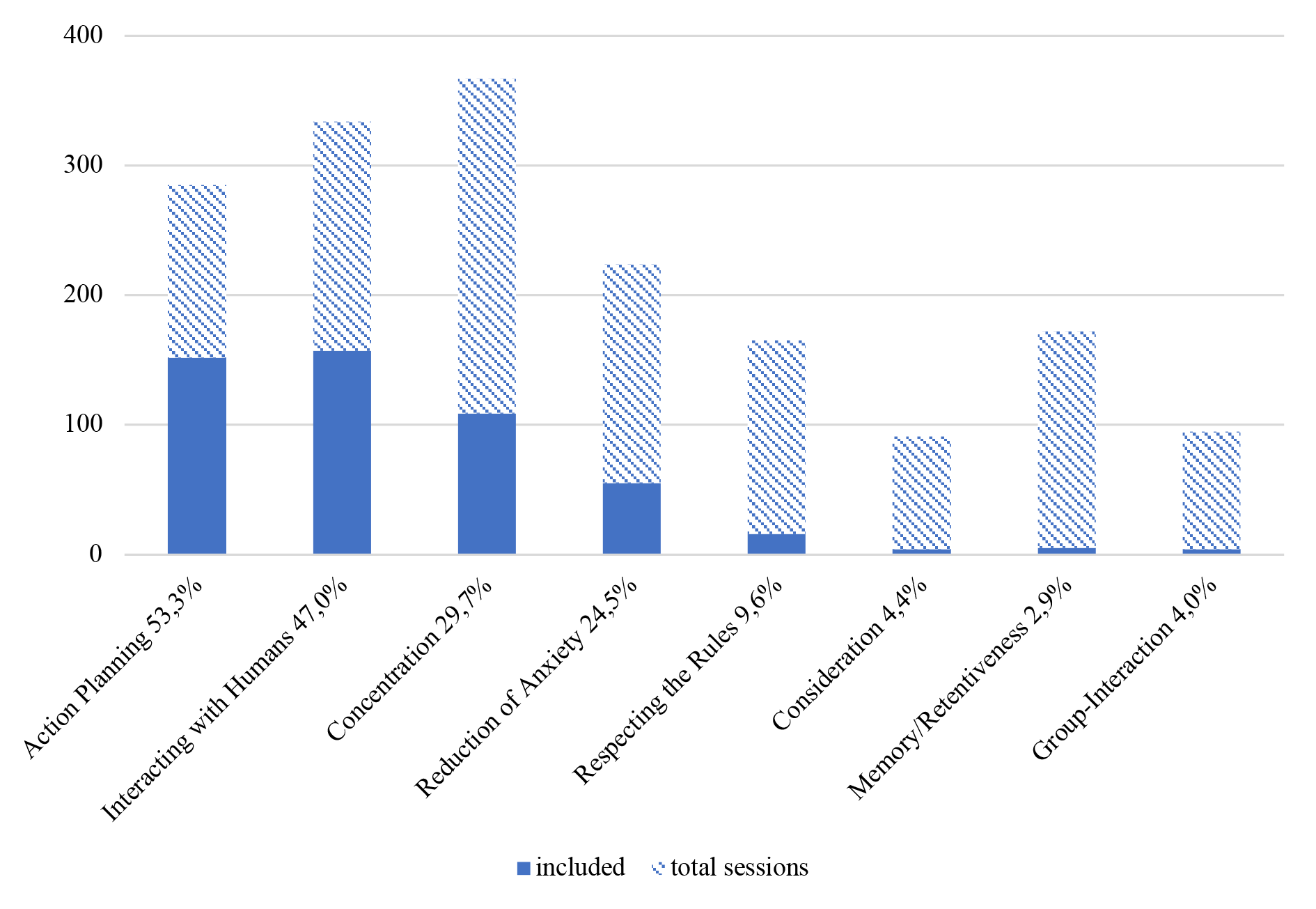

Supplement: S9 Fig — (TIF) [file pone.0320238.s009.tif]
