# Supplementary material for: Psychological aspects of hippotherapy for children with severe neurological impairment: An exploratory study
Source: PLoS One. 2025 Apr 8;20(4):e0320238. doi: 10.1371/journal.pone.0320238 (PMC11978075; doi:10.1371/journal.pone.0320238)
Supplement: S6 Table — (Cerebral Palsy Alliance Research Foundation, 2024, retrieved from: https://cparf.org/what-is-cerebral-palsy/severity-of-cerebral-palsy/gross-motor-function-classification-system-gmfcs/, based on Palisano et al., 1997 [25]. (DOCX) [file pone.0320238.s006.docx]

**S6 Table. Gross Motor Function Classification System (GMFCS).** (Cerebral Palsy Alliance Research Foundation, 2024, retrieved from: <https://cparf.org/what-is-cerebral-palsy/severity-of-cerebral-palsy/gross-motor-function-classification-system-gmfcs/>, based on Palisano et al., 1997 [25].

| GMFCS Level I | - Can walk indoors and outdoors and climb stairs without using their hands for support. - Can run and jump. - Has decreased speed, balance, and coordination. |
| --- | --- |
| GMFCS Level II | - Can walk indoors and outdoors and climb stairs using a railing. - Experiences difficulty with uneven surfaces, inclines, or while in crowds. - Can minimally run or jump. |
| GMFCS Level III | - Walks with assistive mobility devices indoors and outdoors on level surfaces. - May be able to climb stairs using a railing. - May propel a manual wheelchair; may require assistance for long distances or uneven surfaces. |
| GMFCS Level IV | - Walking ability is severely limited, even with assistive devices. - Uses a wheelchair most of the time and may propel their own power wheelchair. - May participate in standing transfers. |
| GMFCS Level V | - Has physical impairments that restrict voluntary movement control and the ability to maintain head and neck position against gravity. - Experiences impairment in all areas of motor function. - Can’t sit or stand independently, even with adaptive equipment. - Can’t independently walk, though may be able to use powered mobility devices. |
