# Supplementary material for: Psychological aspects of hippotherapy for children with severe neurological impairment: An exploratory study
Source: PLoS One. 2025 Apr 8;20(4):e0320238. doi: 10.1371/journal.pone.0320238 (PMC11978075; doi:10.1371/journal.pone.0320238)
Supplement: S7 Table — (DOCX) [file pone.0320238.s007.docx]

**S7 Table. Frequency of assigned predefined neuropsychosocial therapy goals.**

| **Predefined therapeutic goal** | **Number of assignments** |
| --- | --- |
| Group interaction | 143 |
| Concentration | 115 |
| Interaction with humans | 110 |
| Action planning/problem-solving | 99 |
| Reduction of anxiety | 71 |
| Respecting the rules | 57 |
| Memory/retentiveness | 51 |
| Showing consideration | 36 |
