# Supplementary material for: Psychological aspects of hippotherapy for children with severe neurological impairment: An exploratory study
Source: PLoS One. 2025 Apr 8;20(4):e0320238. doi: 10.1371/journal.pone.0320238 (PMC11978075; doi:10.1371/journal.pone.0320238)
Supplement: S8 Table — The total number of sessions refers to the sessions by all the patients who had this goal. Excluded sessions report the number of sessions excluded due to the lack of information on the goal. The achieved column shows the ratio of positive and negative behavior in %. (DOCX) [file pone.0320238.s008.docx]

**S8 Table: Ratio of achieved predefined neuropsychosocial goals in hippotherapy sessions.**

| **Predefined goal** | **Total number of sessions** | **Excluded sessions** | **Achieved** |
| --- | --- | --- | --- |
| Action planning/problem-solving | 285 | 133 | 88.7% |
| Interaction with humans | 344 | 177 | 84.6% |
| Group interaction | 443 | 425 | 81.2% |
| Memory/retentiveness | 172 | 167 | 80.0% |
| Showing consideration | 91 | 87 | 66.6% |
| Reduction of anxiety | 224 | 169 | 48.1% |
| Respecting the rules | 165 | 149 | 42.8% |
| Concentration | 367 | 258 | 39.2% |

The total number of sessions refers to the sessions by all the patients who had this goal. Excluded sessions report the number of sessions excluded due to the lack of information on the goal. The achieved column shows the ratio of positive and negative behavior in %.
