# Supplementary material for: Psychological aspects of hippotherapy for children with severe neurological impairment: An exploratory study
Source: PLoS One. 2025 Apr 8;20(4):e0320238. doi: 10.1371/journal.pone.0320238 (PMC11978075; doi:10.1371/journal.pone.0320238)
Supplement: S10 Table — Presence of the family is grouped under positive behavior. Percentages are calculated attains the ntotal sessions = 1142. (DOCX) [file pone.0320238.s010.docx]

**S10 Table. Documented reactions of patients during hippotherapy sessions.**

| **Reaction category** | **Positive reaction (*n* (%))** | **Negative reaction (*n* (%))** |
| --- | --- | --- |
| Had fun | 929 (81.1) | 26 (2.2) |
| Action planning/problem-solving | 567 (49.5) | 45 (3.9) |
| Interaction with humans | 458 (40.0) | 121 (9.8) |
| Motivation | 358 (31.2) | 38 (3.5) |
| Interaction with horses | 334 (29.1) | 11 (0.9) |
| Helping | 268 (23.4) | 10 (0.8) |
| Reduced anxiety | 113 (9.8) | 117 (10.2) |
| Concentration | 106 (9.2) | 138 (12.0) |
| Presence of the family | 80 (7.0) | 24 (2.0) |
| Patience/relaxation | 64 (5.6) | 53 (4.6) |
| Group interaction | 42 (3.7) | 5 (0.4) |
| Memory/retentiveness | 32 (2.7) | 6 (0.5) |
| Showed consideration | 29 (2.5) | 18 (1.6) |
| Respected the rules | 20 (1.7) | 27 (2.3) |

Presence of the family is grouped under positive behavior. Percentages are calculated attains the *n*_total sessions_=1142.
